# The effect of methylation on the let-7-BCL2L1-BCL2 axis and the potential use of hypomethylating and BH3 mimetic drugs in histiocytic neoplasms

**DOI:** 10.1038/s41375-024-02459-5

**Published:** 2024-11-08

**Authors:** Mali Salmon-Divon, Refael Meyuchas, Ofer Shpilberg, Elimelech Okon, Jamal Benhamida, Mariko Yabe, Kseniya Petrova-Drus, Tal Zvida-Bloch, May Basood, Roei Mazor, Benjamin H. Durham, Julien Haroche, Omar Abdel-Wahab, Eli L. Diamond, Oshrat Hershkovitz-Rokah

**Affiliations:** 1 Department of Molecular Biology, Faculty of Natural Sciences, Ariel University, Ariel, Israel; 2 Adelson School of Medicine, Ariel University, Ariel, Israel; 3 Translational Research Lab, Assuta Medical Centers, Tel-Aviv, Israel; 4 Clinic of Histiocytic Neoplasms, Institute of Hematology, Assuta Medical Center, Tel-Aviv, Israel; 5 Faculty of Medicine, Tel Aviv University, Tel Aviv, Israel; 6 Department of Pathology and Laboratory Medicine, Memorial Sloan Kettering Cancer Center, New York, NY USA; 7 Molecular Pharmacology Program, Sloan Kettering Institute, Memorial Sloan Kettering Cancer Center, New York, NY USA; 8 Service de Médecine Interne, Hôpital Universitaire Pitié Salpêtrière-Charles Foix, Sorbonne Université, Faculté de Médecine, Paris, France; 9 Department of Neurology, Memorial Sloan Kettering Cancer Center, New York, NY USA

**Keywords:** Cell signalling, Myeloproliferative disease

Histiocytic neoplasms are a rare group of diseases with significant morbidity due to histiocytic infiltration to critical organ systems, impeding their normal functions. Diagnosis is based on the identification of CD68+ histiocyte infiltration within tissues, demonstrating varied expression of dendritic and monocytic lineage cells depending on the type of histiocytosis [1, 2]. The predominant histiocytoses are Erdheim-Chester disease (ECD), Langerhans cell histiocytosis (LCH), and Rosai-Dorfman-Destombes (RDD) disease. The pathogenesis of histiocytic neoplasms is driven by inflammation [1] and mutations mainly activating the MAPK/ERK [3] and PI3K pathways [1, 3, 4], but little is known about the epigenetic landscape involved in these neoplasms.

Epigenetic changes, such as DNA methylation, are critical for mammalian development and are frequently implicated in oncogenesis. The process of DNA methylation involves DNA methyltransferases (DNMTs) catalyzing the transfer of a methyl group to a cytosine residue, resulting in the formation of 5-methylcytosine within a CpG dinucleotide. Regions enriched in CpG, called CpG islands, are commonly found in the promoters of genes. In cancer, aberrant hypermethylation of CpG islands is strongly associated with gene silencing, contributing to the inactivation of tumor suppressor genes [5]. Epigenetic changes are potentially reversible by pharmacological inhibition of DNA methylation and histone deacetylation, thus providing a promising target for therapeutic intervention.

Given that histiocytic neoplasms have predominantly been analyzed for DNA mutations, profiling their methylome can enhance our understanding of disease pathogenesis.

Moreover, pharmacological inhibition of BRAF and MEK has shown clinical promise in histiocytic neoplasms; however, their clinical applicability is limited due to toxicity profile, side effects and documented resistance [6, 7]. Therefore, identifying new therapeutic targets is needed for these patients.

To characterize the epigenetic landscape of histiocytic neoplasms, we conducted a methylation profile of LCH (*n* = 14), ECD (*n* = 6), and RDD patients (*n* = 10) from tissue biopsies. These profiles were compared to the methylation profile of controls, suture granulomas (*n* = 4), i.e. localized inflammatory reactions characterized by non-neoplastic histiocytic infiltrates. Supplementary Table 1 shows the patient’s characteristics. Principal component analysis (PCA) revealed distinct clustering, with control samples separating significantly from patients’ samples (Fig. 1A). In total, we identified 23,322 differentially methylated positions (DMPs) (FDR < 0.05 and | Δβ | ≧ 20%). The distribution of hypo- and hyper-DMP is shown in Supplementary Fig. 1. Further analysis identified 1832 differentially methylated regions (DMRs) with *p*-value cutoff of 0.05 and a methylation difference of at least 0.1. The methylation profile was not influenced by age, gender, disease site, or mutation type (Supplementary Fig. 2A–D). Additionally, since the control DNA samples were derived from skin, we compared only the disease sites (excluding controls). The patients’ skin biopsies did not form a separate cluster from other organs but were instead dispersed throughout the dataset (Supplementary Fig. 2E). To elucidate the function of methylated regions, we performed GO pathway analysis to annotate genes associated with each differentially methylated region. This analysis revealed that the DMRs are involved in inflammatory response, RAS, TNF and PI3K-mTOR signaling pathways (Fig. 1B), known to be implicated in histiocytic neoplasms [3, 8, 9]. Specifically, GO analysis indicated that these DMRs may have a role in pathways related to the regulation and generation of non-coding RNAs (Supplementary Fig. 3). Subsequent bioinformatics methylation analysis identified prominent hypermethylated sites in histiocytosis patients within the MIRLET7BHG, the DNA transcript which generate the let-7b miRNA (Supplementary Table 2 and Fig. 2A). Methylation of these sites was previously shown to have a biological effect, through bisulfite-PCR analysis, influencing the expression of mature let-7b [10]. Given that miRNAs are initially transcribed from DNA and subsequently processed and cleaved by a complex machinery to generate mature miRNAs, we analyzed let-7b mature miRNA levels in ECD, LCH, RDD, and healthy control plasma samples. We observed a significant downregulation of let-7b expression (Fig. 2B), suggesting a potential mechanism for the silencing of let-7b in these diseases.

**Fig. 1:**
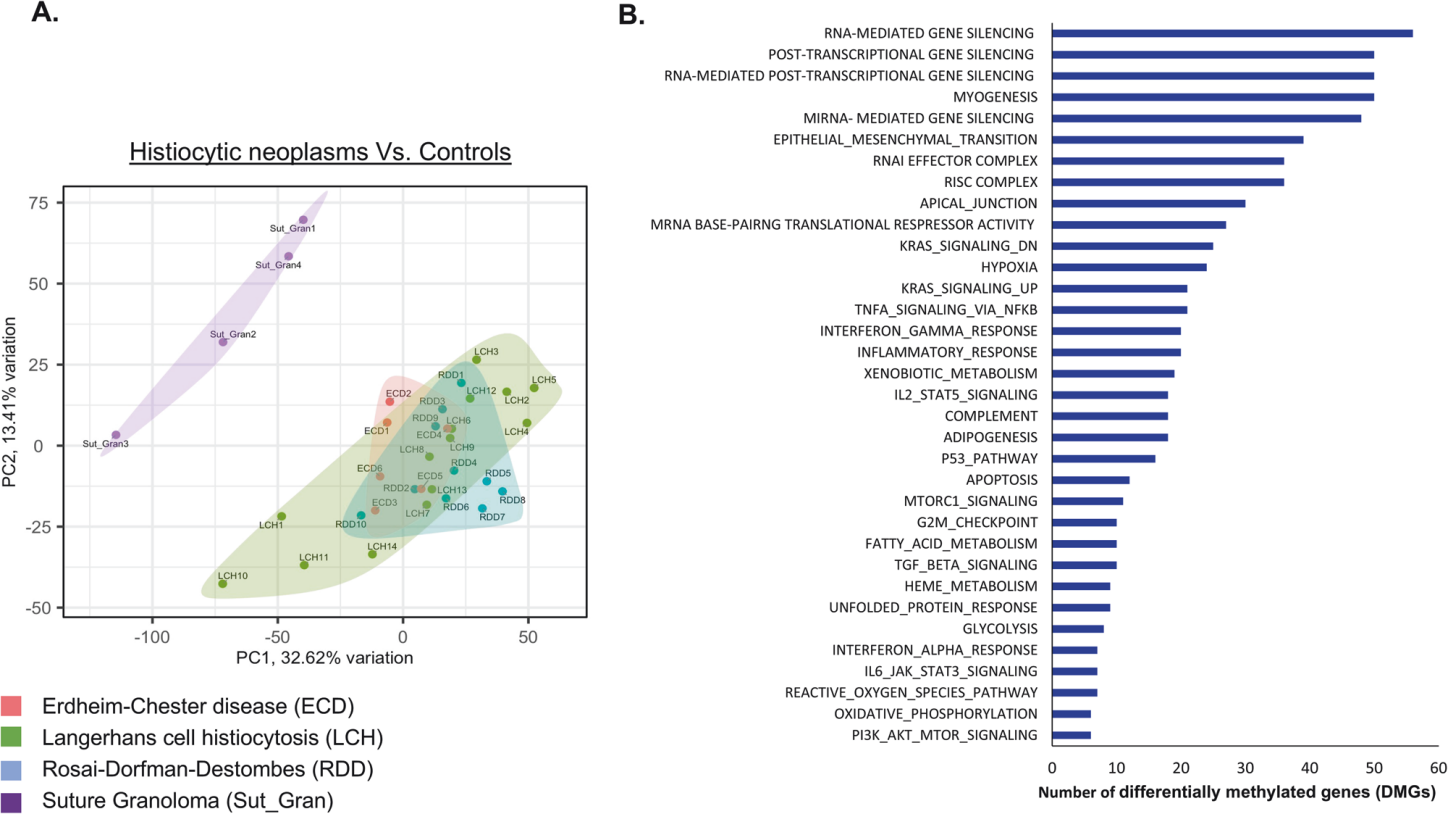
Methylation profile of histiocytic neoplasms. **A** Principal component analysis (PCA) mapping of Illumina methylation EPIC/850k bead array. PCA analysis confirmed a differential methylation pattern between controls (Suture Granulomas, Purple), ECD (Red), LCH (Green) and RDD (Blue) tissue biopsies. **B** Enriched GO for biological pathways, on the top of the list pathways related to regulation and generation of non-coding RNAs.

**Fig. 2:**
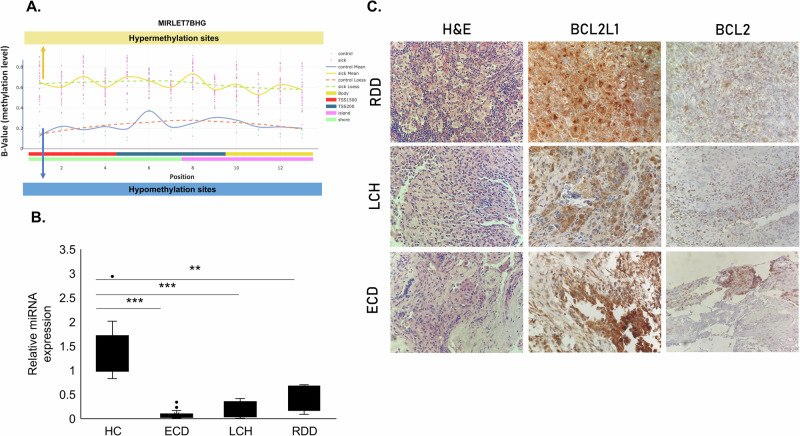
Methylation levels of the MIRLET7B host gene, let-7b miRNA expression, BCL2L1 and BCL2 expression. **A** Methylation levels (β value) plotted against the genomic position of the CpG sites within the differentially methylated region (DMR) associated with the let-7b host gene. The graph illustrates the average methylation levels in controls (blue line) compared to the average methylation levels observed in histiocytosis neoplasms (ECD, LCH, and RDD) displayed as an orange line. **B** let-7b miRNA expression in plasma samples of healthy controls (HC, *n* = 10), ECD (*n* = 25), LCH (*n* = 7), and RDD (*n* = 8) patients by qRT-PCR, normalized to spike-in control cel-miR-39. **C** BCL2L1 is highly expressed in tissue biopsies from histiocytosis patients by IHC. A representative stain from one RDD, one LCH, and one ECD patient. Additionally, BCL2 is slightly overexpressed is these patients (X400). **p < 0.01, ***p < 0.001.

As miRNAs can regulate gene expression by binding to complementary sequences of their target genes, we conducted a screening for let-7b targets utilizing bioinformatic tools (TargetScan and Qiagen IPA analysis tool [11]). Notably, one of the validated targets identified was the anti-apoptotic protein BCL2L1, also known as BCL-XL [12]. We assessed the mRNA expression of BCL2L1 in tissue biopsies from ECD patients as compared to tissues of healthy donors obtained by post-mortem autopsy. We found that ECD patients have elevated mRNA levels of BCL2L1 (Supplementary Fig. 4). Next, we analyzed the expression of BCL2L1 protein levels, by immunohistochemistry, in tissue biopsies from 21 samples (ECD *n* = 7, LCH *n* = 7, and RDD *n* = 7). All the biopsies were tested for histiocytosis markers as part of the routine clinical diagnostic procedure (data not shown). BCL2L1 protein expression was elevated in 100% (21/21) of patients (Fig. 2C and Supplementary Fig. 5). Given the functional relationship between BCL2L1 and the BCL2 oncoprotein, we also measured BCL2 protein expression, which was found to be positive in 52% (11/21) of the patients (Fig. 2C and Supplementary Fig. 5). Hogstad et. al. have reported increased BCL2L1 expression in LCH [13]. Our data support this finding in an independent cohort of 14 LCH patients and, for the first time, demonstrated elevated BCL2L1 expression in ECD and RDD patients.

As the let-7b host gene showed hypermethylation, we used the Ba/F3 cells stably overexpressing the BRAF^V600E^ mutation and treated them with the hypomethylating agent 5-Azacitidine. This treatment resulted in the overexpression of let-7b miRNA (Supplementary Fig. 6A), downregulation of BCL2L1 protein levels (Supplementary Fig. 6B), and induction of apoptosis (Supplementary Fig. 6C), supporting our hypothesis regarding the methylation and regulation on this specific axis. Furthermore, treatment with BH3 mimetic drugs, specifically the BCL2 inhibitor (venetoclax) and the BCL2L1 inhibitor (WEHI-539), led to increased levels of apoptosis (Supplementary Fig. 6D, E).

This discovery holds clinical implications, as treatment with BH3 mimetic drugs may potentially reverse the disease phenotype by targeting anti-apoptotic proteins.

A limitation of this experiment is the absence of direct DNA methylation analysis to confirm the effect of 5-Azacytidine on the let-7b promoter. Other epigenetic mechanisms, such as histone modifications, may also contribute to the silencing of let-7b, and 5-Azacytidine may have broader, indirect effects on gene expression beyond DNA demethylation. Additionally, post-transcriptional regulation could influence the observed changes in let-7b levels. Finally, we acknowledge the limitation that our samples are heterogeneous, which reflects the rarity of these diseases, and the challenges associated with collecting large-scale homogeneous samples in histiocytosis studies.

In conclusion, our study demonstrates that epigenetic regulation is likely an additional driver of histiocytic neoplasms. We have previously reported downregulation of the let-7 family members in histiocytic neoplasms due to the regulation of MAPK signaling [14, 15].

This study suggests that methylation of the let-7b gene contributes to its aberrant expression, which may be crucial to disease pathogenesis.

The findings highlight the importance of DNA methylation in regulating the expression of non-coding molecules, leading to the upregulation of their targets, and offering new avenues for therapeutic intervention. The potential use of hypomethylating agents and BH3 mimetic drugs represents a promising strategy for treating histiocytic neoplasms, warranting further clinical investigation.

## Supplementary information

Supplementary Methods Tables and figures

## Data Availability

The data supporting the findings of this study are available in GEO database under the accession number GSE279030.
