# Supplementary material for: The effect of methylation on the let-7-BCL2L1-BCL2 axis and the potential use of hypomethylating and BH3 mimetic drugs in histiocytic neoplasms
Source: Leukemia. 2024 Nov 8;39(2):516–9. doi: 10.1038/s41375-024-02459-5 (PMC11794134; doi:10.1038/s41375-024-02459-5)
Supplement: Supplementary file 1 — Supplementary Methods Tables and figures [file 41375_2024_2459_MOESM1_ESM.docx]

**Material and Methods**

**Methylation profile and bioinformatics analysis**

Methylation profiles of six ECD, 14 LCH, and 10 RDD patients were analyzed from diagnostically confirmed tumor biopsies (treatment-naïve) by Illumina EPIC-methylation array according to the manufacturer protocol. These were compared to controls, suture granulomas (n=4), localized inflammatory reactions characterized by non-neoplastic histiocytic infiltrates. Suture granulomas represent a localized inflammatory response to foreign material and share several biological processes with histiocytic disorders, including macrophage activation and granuloma formation. These shared mechanisms make suture granulomas a relevant comparative model for examining the molecular pathways involved in immune activation and tissue response. Additionally, the non-neoplastic nature of suture granulomas provides a valuable contrast to histiocytic neoplasms, allowing for the differentiation of molecular pathways associated with the pathological proliferation of histiocytes in histiocytosis versus the regulated, reactive processes observed in granuloma formation. All samples from both the control and patient groups contained >50% histiocyte content within a macrodissected region. Histiocytic purity was quantified by a pathologist’s microscopic examination.

Quality control of methylation data was conducted using the R package ChAMP (v2.30) (1) based on the following exclusion criteria: 1) probes with a detection p value above 0.01, 2) probes with fewer than three beads in at least 5% of samples per probe, 3) all non-CpG probes, 4) all single nucleotide polymorphism (SNP)-related probes, 5) all multihit probes, and 6) all probes located in chromosomes X and Y. Based on these criteria, 732,264 CpG sites were included in the study. Beta values were then normalized using the Beta Mixture Quantile dilation (BMIQ) method (2). After normalization, the expected bimodal beta value distribution was observed for all samples. Singular value decomposition (SVD) analysis (3) was carried out to estimate both technical and biological sources of variation in our experiments. The champ.SVD function with default parameters was used for this analysis, with covariates set as follows: sample group (ECD/LCH/RDD/Control), sample type (Sick/Control), array, slide, sample plate. Principal Component Analysis (PCA) was applied using PCAtools (v.2.12) to the normalized methylation dataset, after excluding 10% of variables based on low variance, to explore relations among the different samples.

Differentially methylated probes (DMPs) were identified using the "limma" package (v.3.56) (4), which calculates the p-value for differential methylation based on a linear model. To control the false positive rate due to multiple comparisons, the Benjamini and Hochberg false discovery rate (FDR) method was applied. The delta beta value represents the average difference in beta values between two groups. DMPs were considered significant if they had an adjusted p-value < 0.05 and a delta beta > 0.2. The data has been uploaded to the GEO database under the accession number GSE279030.

Differentially methylated regions (DMRs) were identified using the DMRcate algorithm (5) using log2 transformed beta-values (M-values). We applied a smoothing parameter (lambda=1000) and a scaling factor (C=2) to detect DMRs, ensuring that clusters within 1000 base pairs were considered. Regions required a minimum of 7 CpG sites (min.cpgs = 7) and a beta value difference of at least 0.1 (betacutoff = 0.1) to be deemed significant. We used the missMethyl R package (v1.34) (6) to evaluate the identified DMRs for significant gene and pathway enrichments, employing all CpGs as the background. This analysis utilized Wallenius' noncentral hypergeometric test. For Gene Ontology (GO) and Kyoto Encyclopedia of Genes and Genomes (KEGG) pathway enrichment analyses, we applied the GOregion function of missMethyl. Additionally, the gsaregion function was employed to conduct gene set analysis (GSA) with the Hallmark gene sets from the Molecular Signatures Database (MSigDB) v7.1.

**Quantitative reverse transcription polymerase chain reaction (qRT-PCR)**

MiRNA from cells and plasma samples were isolated using the RNeasy Plus Kit (Qiagen, Germany) and miRNeasy Serum/Plasma kit, respectively. Isolation procedure and miRNA reverse-transcription (RT) with miRNA specific stem-looped RT primers (Life Technologies, Thermo Fisher Scientific Inc.) were previously describe (7, 8). The expression of the miRNAs was quantified relative to the expression of spike-in control cel-miR-39 (Assay ID #200) for plasma samples, and RNU48-snRNA (Assay ID #1006) for cell lines as internal controls (Applied Biosystems; Thermo Fisher Scientific Inc.).

For gene expression assay, RNA was isolated using the RNeasy Plus Kit (QIAGEN, Germany) and reverse transcribed using the high-capacity cDNA RT kit (Life Technologies; Thermo Fisher Scientific Inc.). cDNA was equally pre-amplified and qRT-PCR reaction conditions for BCL2L1 (Assay ID #Hs00236329_m1, Applied Biosystems; Thermo Fisher Scientific Inc) were the same as previously published (9). HPRT1 (Assay ID #Hs99999909_m1, Applied Biosystems; Thermo Fisher Scientific Inc) was used as endogenous control.

**Immunohistochemistry (IHC)**

Immunohistochemical staining was performed on 4µm Formalin-Fixed Paraffin-Embedded (FFPE) sections using the Leica Bond max system (Leica Biosystems Newcastle Ltd, UK). Sections were dewaxed and pretreated for 5 minutes with epitope-retrieval solution (ER2, Leica Biosystems Newcastle Ltd, UK) followed by 30 minutes incubation with BCL2L1 antibody (1:750, #10783, by Proteintech, Rosemont, IL, USA). The Leica Refine HRP kit (Leica Biosystems Newcastle Ltd, UK) used for detection and counter-stain with Hematoxylin.

**Compounds**

The following inhibitors were dissolved in dimethyl sulfoxide (DMSO): 5- Azacitidine-2-deoxycytidine [(5-Aza) hypomethylating agent, 11164, Cayman chemical, Ann Arbor, Michigan, USA], Venetoclax (BCL2 inhibitor, V-3579, LC Laboratories, MA, USA), WHEI-539 (BCL2L1 inhibitor, MedChem Express, NJ, USA).

**Western blot**

Cells were harvested using RIPA lysis buffer (Sigma Aldrich, USA) supplemented with protease inhibitor cocktail (Thermo Fisher Scientific Inc.). Equal amounts of protein were separated on a 10% SDS-PAGE gel (Bio-Rad, USA) and blotted onto nitrocellulose membranes. Membranes were blocked and incubated with the following primary antibodies: BCL2L1 (#2764) and Tubulin (#3873) (Cell signaling technology, MA, USA). Secondary antibodies goat anti mouse and goat anti rabbit were purchased from Li-Cor Biosciences, USA.

**Apoptosis assay**

Cells were assayed using the Annexin V apoptosis detection kit/phycoerythrin (PE), 7-aminoactinomycin D (7-AAD) (eBioscience, Inc., Thermo Fisher Inc., #88-8102-74) according to the manufacturer's protocol. Cells were treated with the appropriate drug. After 72 hours, the cells were harvested, washed in PBS, and then washed again in binding buffer. A total of 5 × 10⁵ cells per 100 µl were resuspended in 100 µl of binding buffer and stained with 5 µl of Annexin V. Samples were incubated for 15 minutes at room temperature, followed by the addition of 5 µl of 7-AAD. Cells were analyzed using the CytoFlex system

**Table 1. Patient's characteristics**

| **Driver**  **mutation** | **Disease**  **site** | **Gender** | **Age** | **Sample** |  |
| --- | --- | --- | --- | --- | --- |
| KRAS G12R, ARAF P216A | Skin | M | 51.2 | ECD1 | **ECD** |
| BRAF V600E | Skin | M | 43.5 | ECD2 |  |
| CBL | Skin | F | 58.9 | ECD3 |  |
| MAP2K1 F53L | Pleura | M | 53.0 | ECD4 |  |
| BRAF V600E | Brain | M | 51.3 | ECD5 |  |
| BRAF-PICALM fusion | Colon | F | 50.3 | ECD6 |  |
| CSF2RA-BRAF fusion | Skin | F | 28.0 | LCH1 | **LCH** |
| MAP2K1 E102_I103del | Soft tissue | M | 20.0 | LCH2 |  |
| BRAF V600E | Bone | F | 30.8 | LCH3 |  |
| MAP2K1 E102_I103del | Bone | M | 22.6 | LCH4 |  |
| MAP2K1 Q58_E62del | Bone | M | 27.6 | LCH5 |  |
| BRAF V600E | Skin | F | 33.9 | LCH6 |  |
| Unknown | Lung | M | 24.8 | LCH7 |  |
| MAP2K1 E102_I103 indel | Brain | M | 29.4 | LCH8 |  |
| BRAF V600E | Muscle | F | 37.3 | LCH9 |  |
| BRAF 486_490 indel | Skin | M | 38.1 | LCH10 |  |
| BRAF 486_490 indel | Skin | M | 35.9 | LCH11 |  |
| BRAF V600E | Bone | M | 32.7 | LCH12 |  |
| BRAF N486_P490del | Lung | F | 55.6 | LCH13 |  |
| BRAF V600E | Skin | F | 70.8 | LCH14 |  |
| KRAS K117N | Breast | F | 53.7 | RDD1 | **RDD** |
| MAP2K1 K57T, KRAS A146P | lymph node | F | 51.0 | RDD2 |  |
| KRAS in-frame insertion Y64S65insVS | Nasopharynx | F | 78.5 | RDD3 |  |
| BRAF 486_490 indel | Brain | M | 66.2 | RDD4 |  |
| KRAS K117N | Lymph node | F | 35.1 | RDD5 |  |
| MAP2K1 C121S | Skin | F | 60.1 | RDD6 |  |
| KRAS K117R | Colon | F | 57.9 | RDD7 |  |
| KRAS A146V | Skin | F | 60.4 | RDD8 |  |
| Unknown | Skin | M | 61 | RDD9 |  |
| Unknown | Skin | F | 29.4 | RDD10 |  |
| ND | Skin | F | 44 | Cont_1 | **Control** |
| ND | Skin | F | 67 | Cont_2 |  |
| ND | Skin | F | 74 | Cont_3 |  |
| ND | Skin | M | 46 | Cont_4 |  |

Abbreviations: ECD, Erdheim-Chester disease; LCH, Langerhans cell histiocytosis;

RDD, Rosai-Dorfman-Destombes; Cont, Controls (Suture Granuloma); ND, Not determine; M, Male; F, Female. Age refers to age at diagnosis.

**Table 2. Methylation sites**

| Feature | Gene | Strand | MAPINFO | CHR | ID |
| --- | --- | --- | --- | --- | --- |
| TSS1500 | MIRLET7BHG | R | 46480891 | 22 | cg04329382 |
| TSS1500 | MIRLET7BHG | F | 46481099 | 22 | cg03412618 |
| TSS1500 | MIRLET7BHG | F | 46481248 | 22 | cg10352237 |
| TSS1500 | MIRLET7BHG | F | 46481603 | 22 | cg21094022 |
| TSS200 | MIRLET7BHG | R | 46481677 | 22 | cg09328012 |
| TSS200 | MIRLET7BHG | F | 46481727 | 22 | cg11538810 |
| TSS200 | MIRLET7BHG | R | 46481785 | 22 | cg26422762 |
| TSS200 | MIRLET7BHG | R | 46481816 | 22 | cg20076516 |
| TSS200 | MIRLET7BHG | F | 46481822 | 22 | cg24731441 |
| Body | MIRLET7BHG | R | 46481948 | 22 | cg11383208 |
| Body | MIRLET7BHG | F | 46482009 | 22 | cg21450723 |
| Body | MIRLET7BHG | F | 46482020 | 22 | cg23408471 |
| Body | MIRLET7BHG | F | 46482023 | 22 | cg07546360 |

**
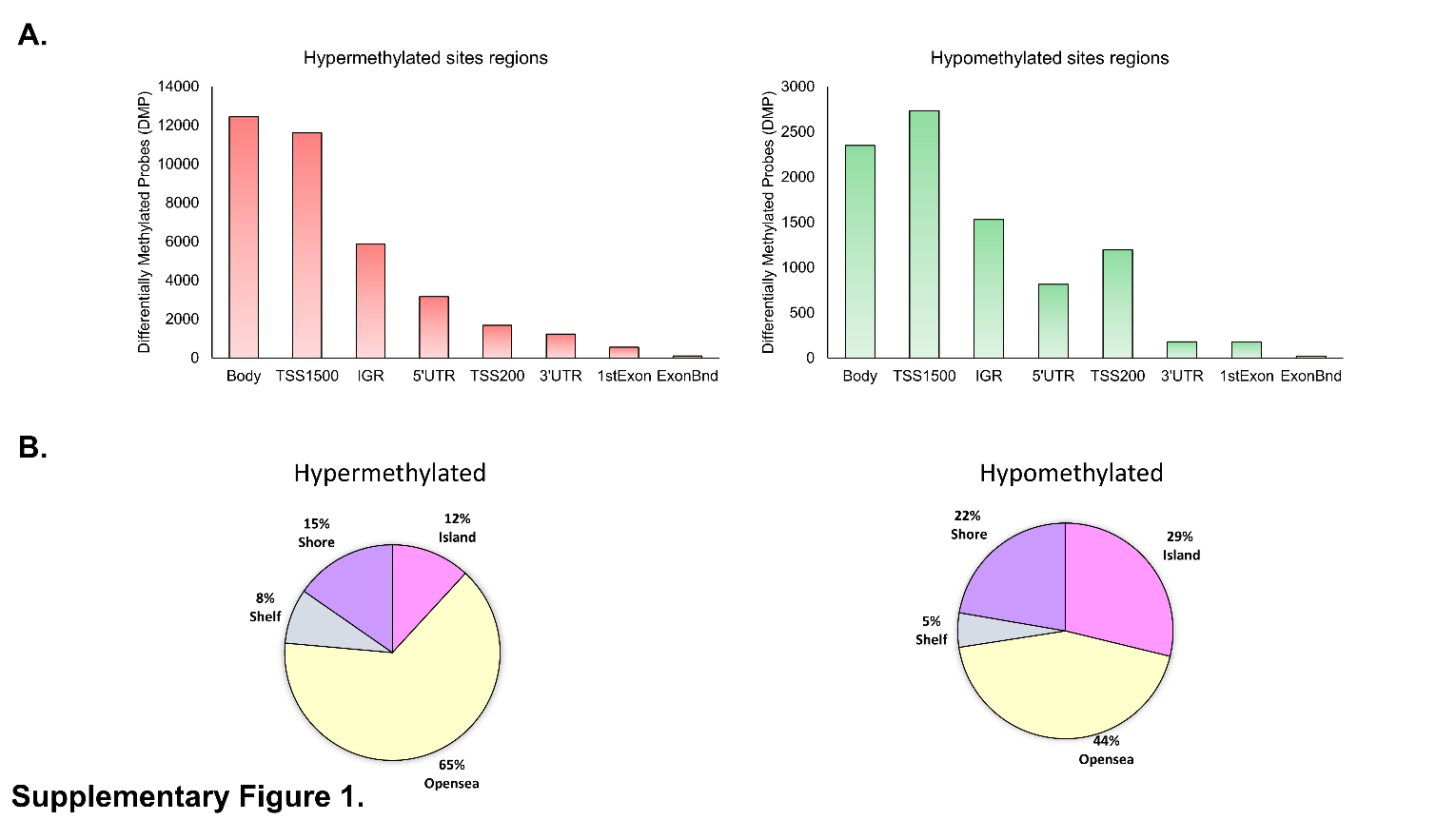
**

**Supplementary Figure 1. Frequency of differentially methylated regions according to functional and CpG island contextual distribution.** **(A)** Bar charts illustrate the distribution frequency of hypermethylated and hypomethylated loci based on their functional positions, including their distance from the transcriptional start site (TSS). **(B)** Percentages of differentially methylated CpG loci located in CpG islands, shores, shelves, or open sea.

**
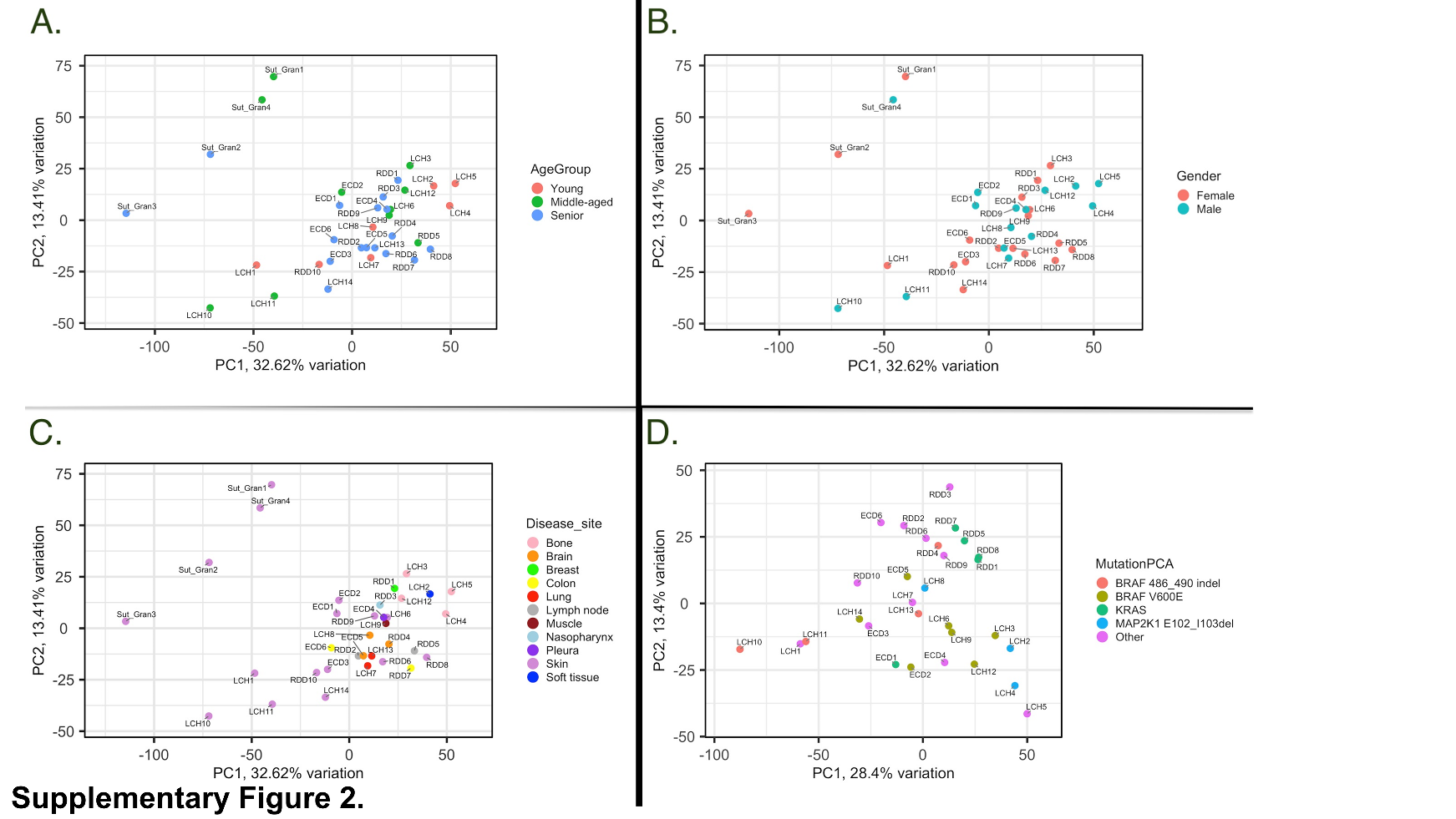
**

**
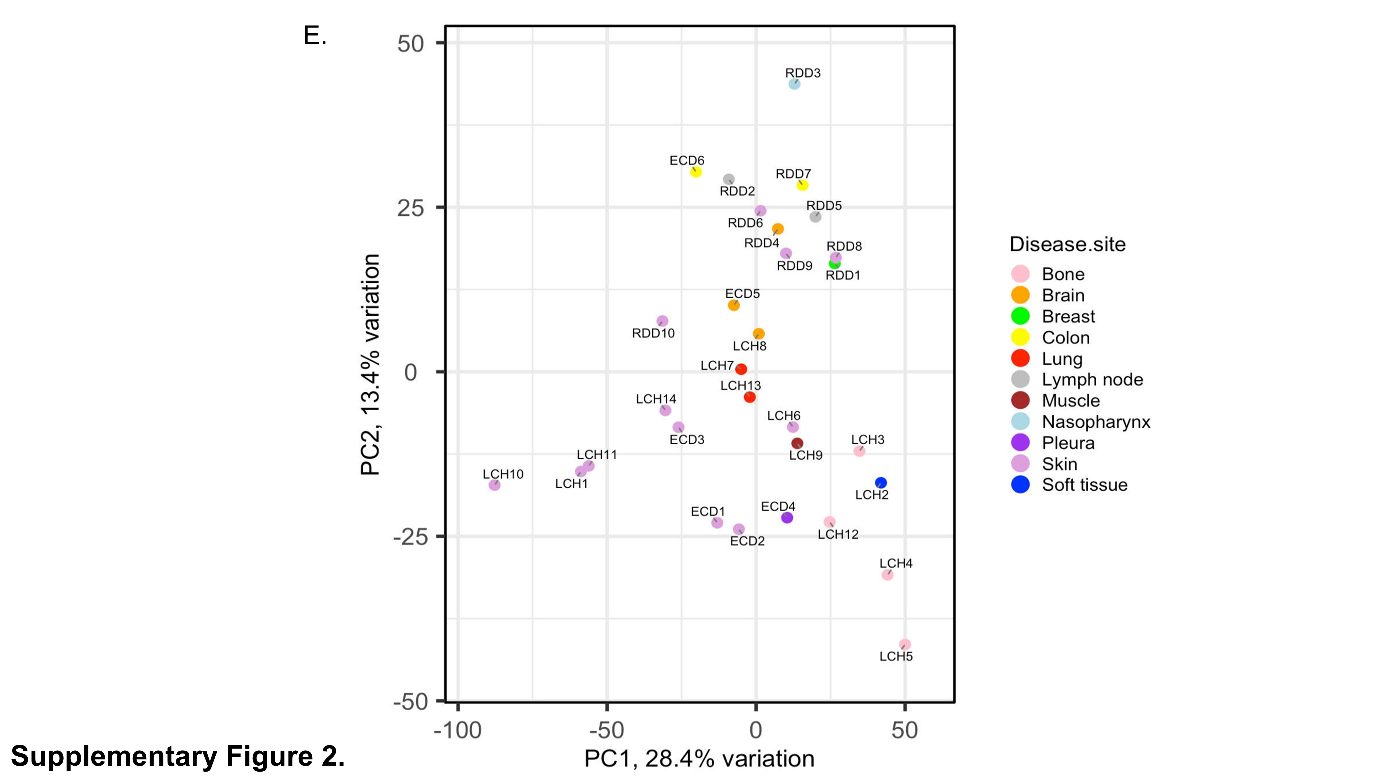
**

**Supplementary Figure 2. Samples clustering by age, gender, disease site and mutation profile**. Principal component analysis (PCA) mapping of Illumina methylation EPIC/850k bead array by **(A)** age (young<30, middle-aged<50, senior>50) **(B)** gender **(C)** Disease site and **(D)** mutation type between controls (Suture Granulomas), ECD, LCH and RDD tissue biopsies. **(E)** PCA mapping of disease site only between patient samples, including ECD, LCH, and RDD tissue biopsies.


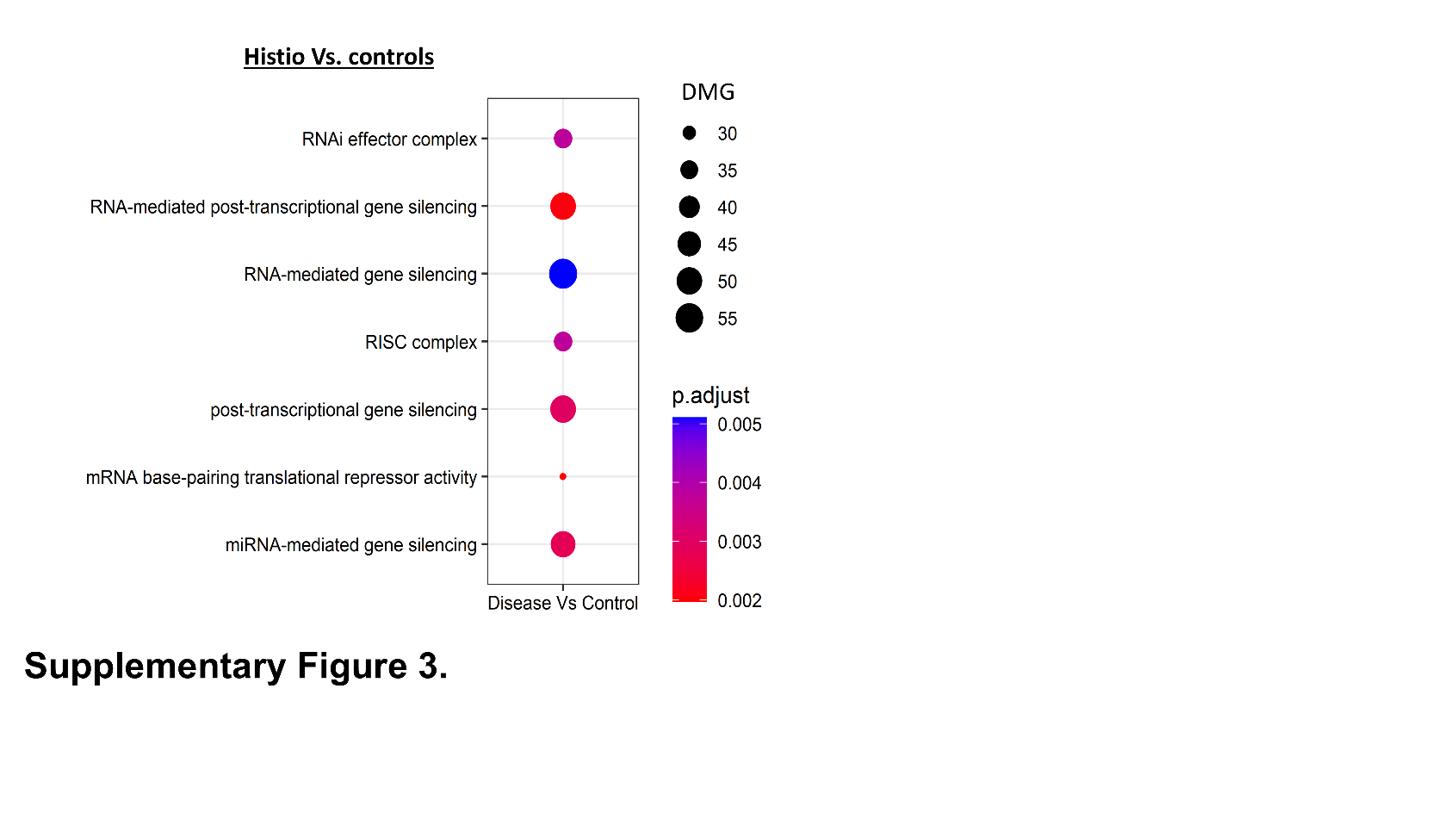


**Supplementary Figure 3. Gene Ontology (GO) analysis for the differentially methylated genes (DMGs).** Focused enriched GO for biological pathways presented in Figure 1B, presenting the top of the list pathways related to regulation and generation of non-coding RNAs. The size of the dots represents the number of DMGs in the significant differentially expressed gene list associated with the GO term and the color of the dots represent the P-adjusted values.


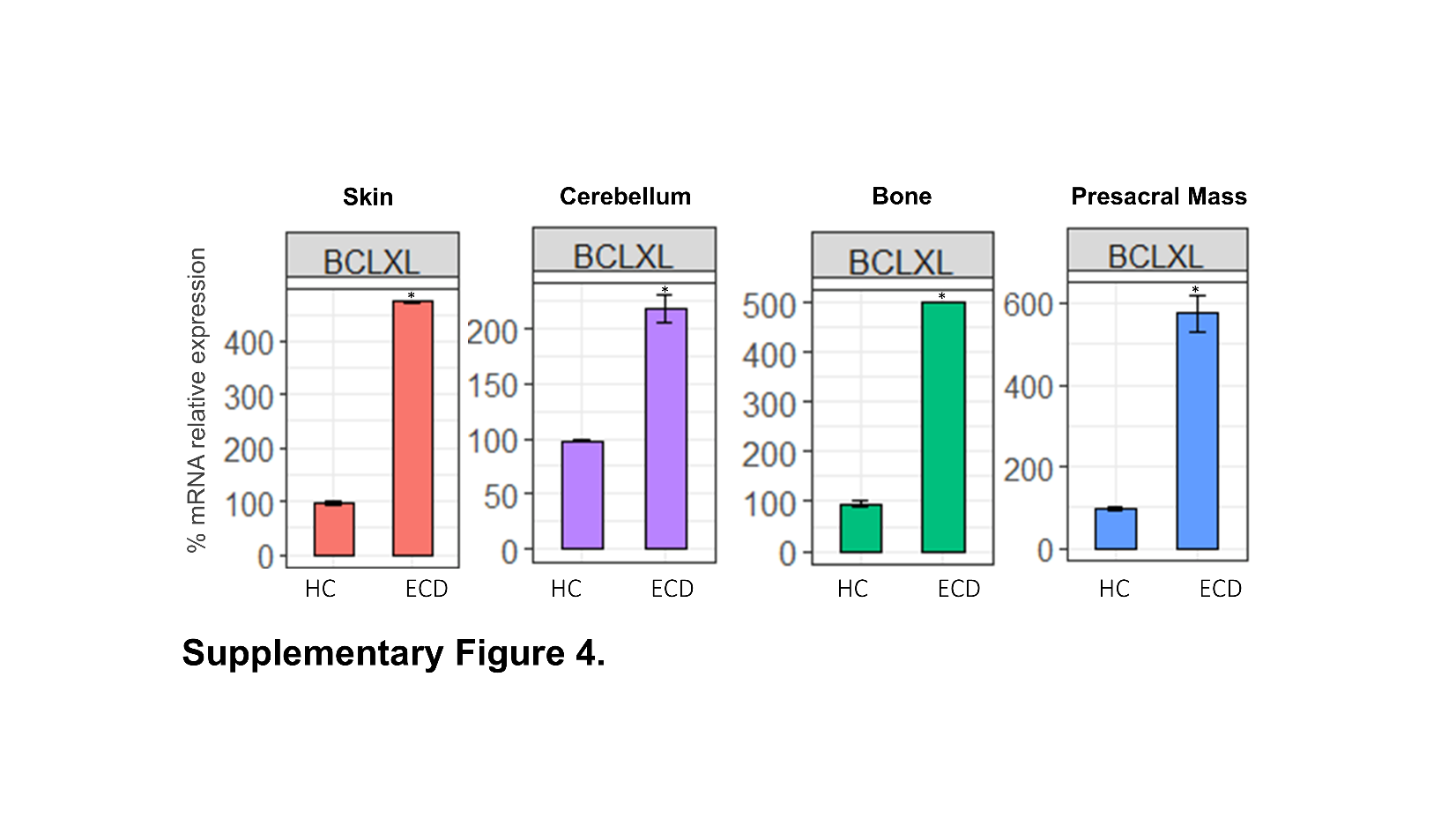


**Supplementary Figure 4.** **BCL2L1 mRNA is highly expressed in ECD tissue samples.** ECD patients (n=3) exhibit high mRNA expression levels of BCL2L1 (BCL-XL) compared to tissues of healthy donors obtained by post-mortem autopsy, as measured by qRT-PCR, normalized to HPRT1. *p < 0.05


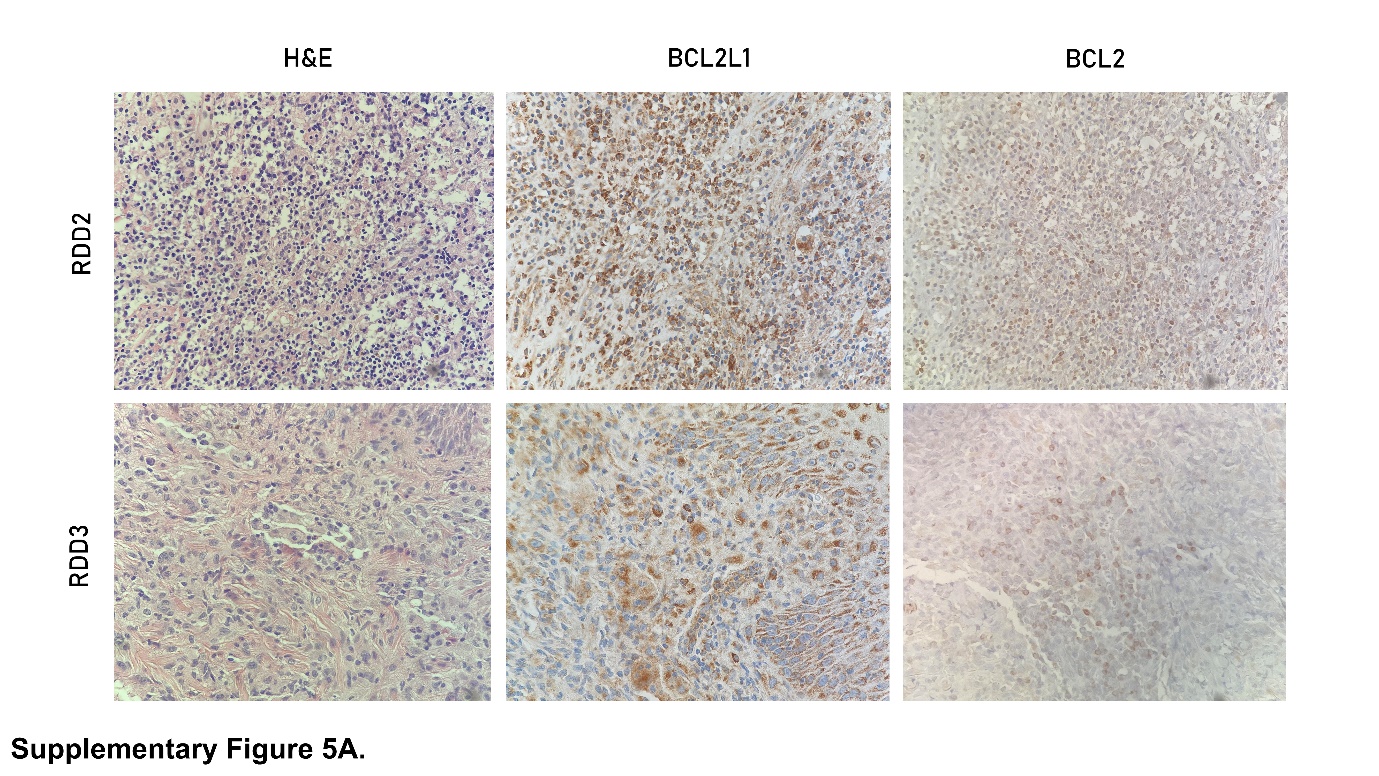

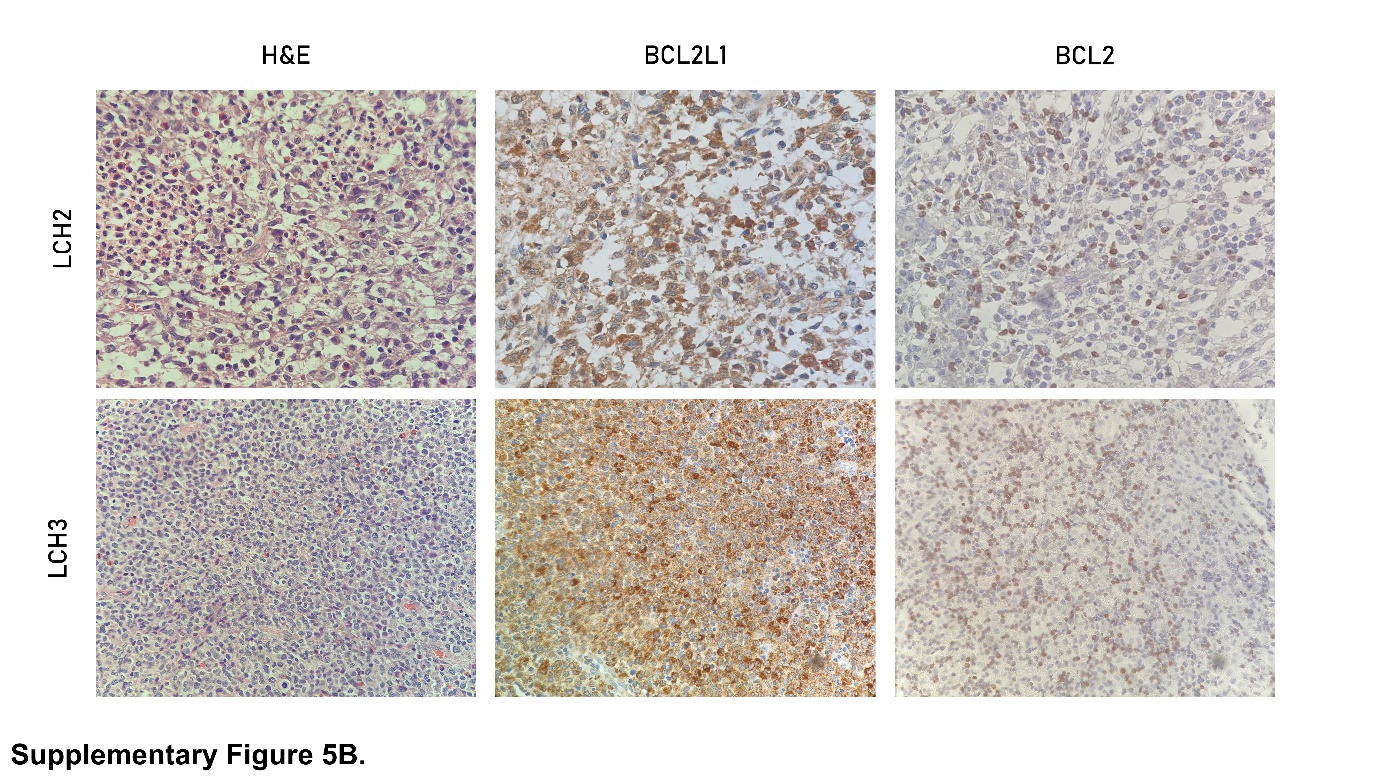


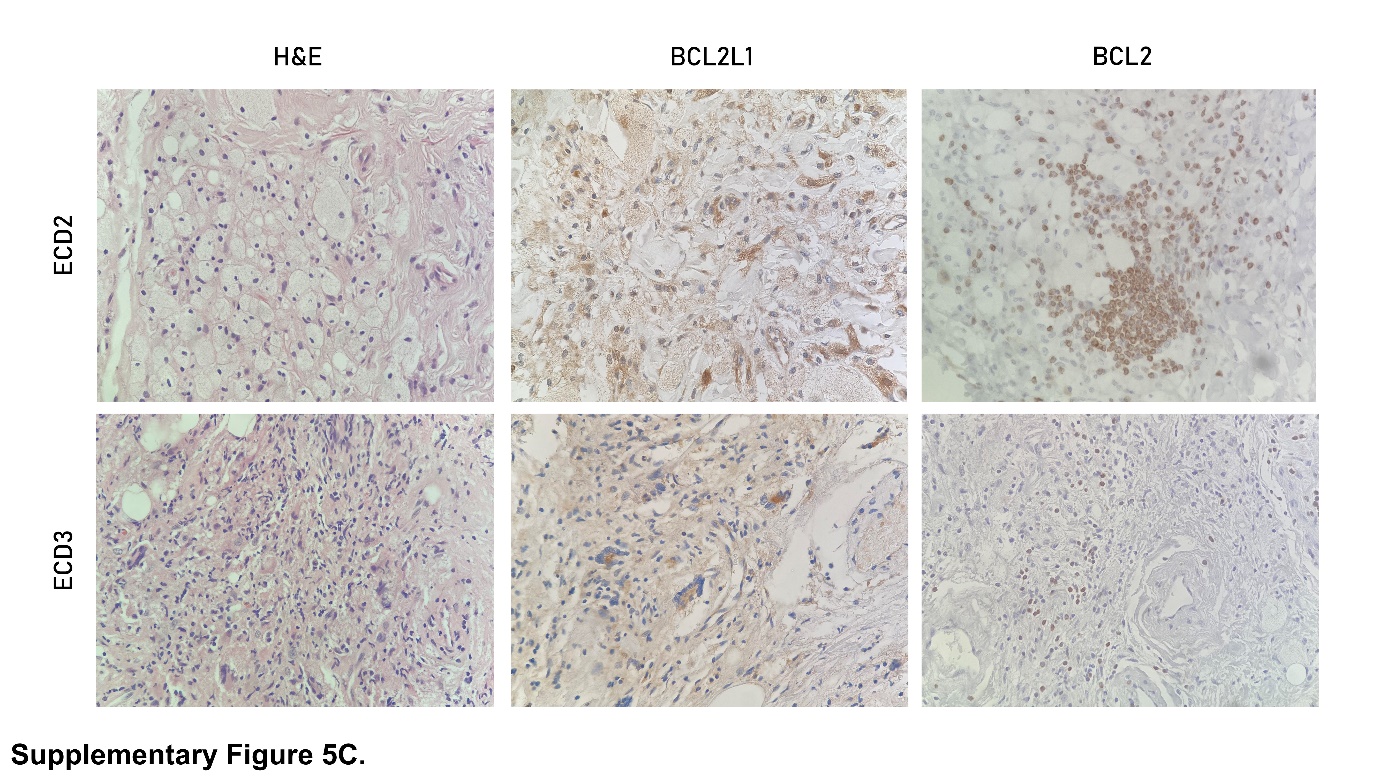


**Supplementary Figure 5. BCL2L1 protein levels in histiocytic neoplasms**. Additional representative stains for BCL2L1 and BCL2 from **(A)** RDD, **(B)** LCH, and **(C)** ECD patients. (X400).


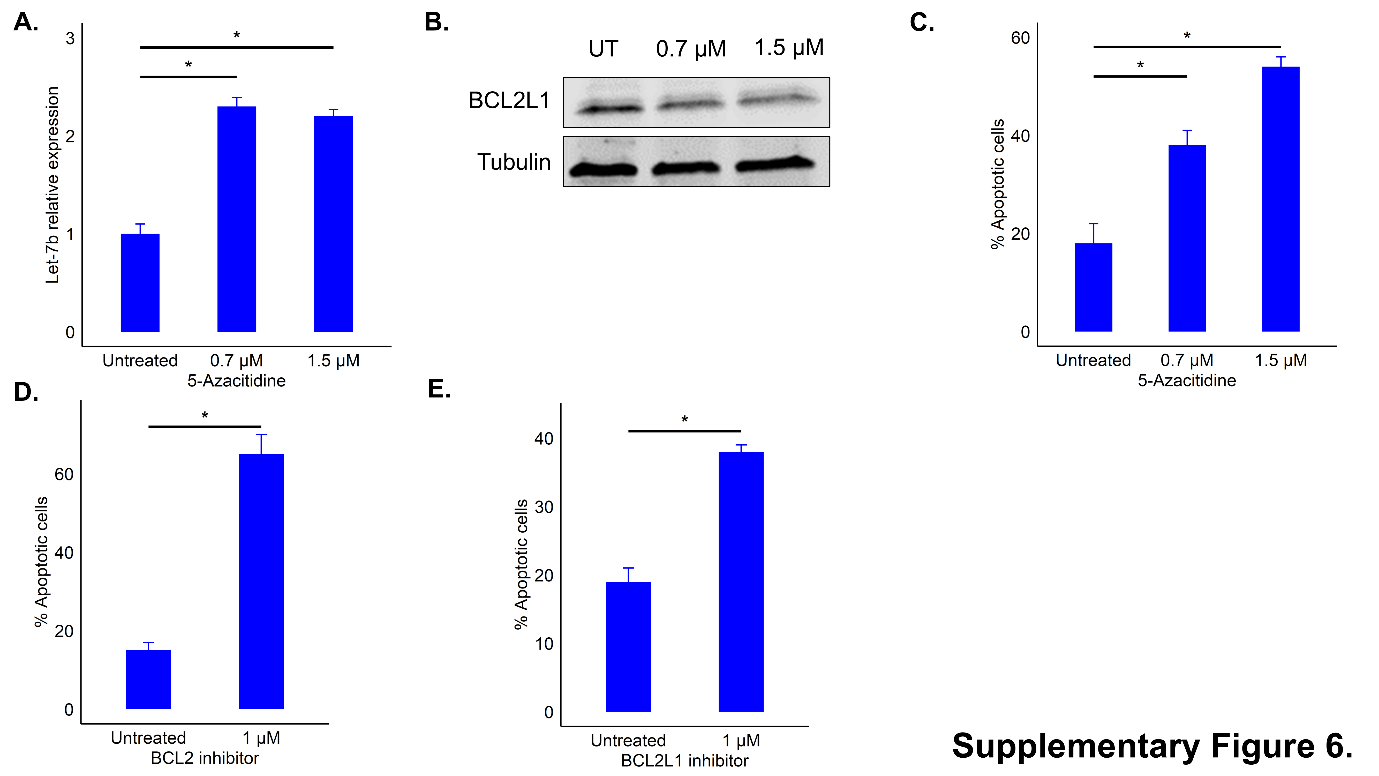


**Supplementary Figure 6. Effect of treatment with hypomethylating agent and BH3 mimetic drugs on BA/F3 BRAF^V600E^ mutant cells. (A)** Upregulation of let-7b expression, measured by qRT-PCR, and **(B)** downregulation of BCL2L1 protein levels (by Western blot) after treatment with the hypomethylating agent (5-AZA) at 0.7 µM and 1 µM for 48h. **(C)** Increased apoptosis levels after treatment with 5-AZA **(D)** BCL-2 inhibitor and **(E)** the BCL2L1 inhibitor as measured by Annexin V positive cells in a flow cytometer. *p<0.05.

**References:**

1. Tian Y, Morris TJ, Webster AP, Yang Z, Beck S, Feber A, et al. ChAMP: updated methylation analysis pipeline for Illumina BeadChips. Bioinformatics. 2017;33(24):3982-4.

2. Teschendorff AE, Marabita F, Lechner M, Bartlett T, Tegner J, Gomez-Cabrero D, et al. A beta-mixture quantile normalization method for correcting probe design bias in Illumina Infinium 450 k DNA methylation data. Bioinformatics. 2013;29(2):189-96.

3. Teschendorff AE, Menon U, Gentry-Maharaj A, Ramus SJ, Gayther SA, Apostolidou S, et al. An epigenetic signature in peripheral blood predicts active ovarian cancer. PLoS One. 2009;4(12):e8274.

4. Ritchie ME, Phipson B, Wu D, Hu Y, Law CW, Shi W, et al. limma powers differential expression analyses for RNA-sequencing and microarray studies. Nucleic Acids Res. 2015;43(7):e47.

5. Peters TJ, Buckley MJ, Chen Y, Smyth GK, Goodnow CC, Clark SJ. Calling differentially methylated regions from whole genome bisulphite sequencing with DMRcate. Nucleic Acids Res. 2021;49(19):e109.

6. Phipson B, Maksimovic J, Oshlack A. missMethyl: an R package for analyzing data from Illumina's HumanMethylation450 platform. Bioinformatics. 2016;32(2):286-8.

7. Weissman R, Diamond EL, Haroche J, Durham BH, Cohen F, Buthorn J, et al. MicroRNA-15a-5p acts as a tumor suppressor in histiocytosis by mediating CXCL10-ERK-LIN28a-let-7 axis. Leukemia. 2022;36(4):1139-49.

8. Weissman R, Diamond EL, Haroche J, Pillar N, Shapira G, Durham BH, et al. The Contribution of MicroRNAs to the Inflammatory and Neoplastic Characteristics of Erdheim-Chester Disease. Cancers (Basel). 2020;12(11).

9. Fishov H, Muchtar E, Salmon-Divon M, Dispenzieri A, Zvida T, Schneider C, et al. AL amyloidosis clonal plasma cells are regulated by microRNAs and dependent on anti-apoptotic BCL2 family members. Cancer Med. 2023;12(7):8199-210.
